# A novel Vi-diphtheria toxoid typhoid conjugate vaccine is safe and can induce immunogenicity in healthy Indonesian children 2–11 years: a phase II preliminary report

**DOI:** 10.1186/s12887-020-02375-4

**Published:** 2020-10-15

**Authors:** Bernie Endyarni Medise, Soedjatmiko Soedjatmiko, Hartono Gunardi, Rini Sekartini, Hindra Irawan Satari, Sri Rezeki Hadinegoro, Angga Wirahmadi, Mita Puspita, Rini Mulia Sari, Jae Seung Yang, Arijit Sil, Sushant Sahastrabuddhe, Novilia Sjafri Bachtiar

**Affiliations:** 1 Department of Child Health, Faculty of Medicine Universitas Indonesia, Dr. Cipto Mangunkusumo General National Hospital, Jalan Diponegoro no, Jakarta, 71 Indonesia; 2 PT. Bio Farma, Bandung, Indonesia; 3 International Vaccine Institute, Seoul, Republic of Korea

**Keywords:** Immunogenicity, Safety, Typhoid conjugate vaccine, Vi-DT vaccine, Vi-PS vaccine

## Abstract

**Background:**

Typhoid fever caused by Salmonella enteric serovar Typhi (*S. Typhi*) is a common cause of morbidity in the world. In 2017, 14.3 million cases of Typhoid and paratyphoid fever occurred globally. School age children between 3 to 19 years old are the most affected. Poor sanitation and multi drug resistance have increased the need for vaccines to reduce the global burden of disease. Based on previous trials, typhoid conjugate vaccines have longer- lasting protection, higher efficacy, require fewer doses and are suitable from infancy that allows them to be incorporated into the routine immunization program. Our previous phase I trial proved that a novel Vi-DT typhoid conjugate vaccine is safe and immunogenic in subjects 2–5 and 18–40 years. Our phase II trial consisted of subjects 6 months to 40 years. Our previously published paper on subjects 6 to < 24 months proved that this vaccine is safe and immunogenic for this age group. Therefore, with this paper we aimed to evaluate the safety and immunogenicity in children 2–11 years.

**Methods:**

A randomized, observer-blind, superiority design of Vi-DT Typhoid conjugate vaccine compared to Vi-polysaccharide vaccine (Vi-PS) phase II study was conducted from October 2018 to December 2018 where 200 subjects aged 2–11 years were recruited. A blood sample prior to vaccination was taken, followed by administration of a single dose of either test vaccine (Vi-DT) or control vaccine (Vi-PS) and then a second blood sample was collected 28 days post vaccination. Adverse reactions were assessed and antibody increment was evaluated at 28 days post vaccination through collected serum sample.

**Results:**

Pain was the most common local reaction. Fever and muscle pain were the most common systemic reactions. Both Vi-DT and Vi-PS groups had roughly the same number of adverse reactions. At 28 days post vaccination, 100% of subjects in the Vi-DT group and 93% of subjects in the Vi-PS group produced antibody increment ≥4 times. The Vi-DT group produced a higher GMT as compared to Vi-PS.

**Conclusion:**

Vi-DT vaccine is safe and immunogenic in children 2–11 years old.

**Trial registration:**

Trial registration number: NCT03460405.

## Background

Enteric fever caused by *Salmonella enterica* serovar Typhi (*S*.Typhi) and *Salmonella enterica* serovar Paratyphi (*S*. Paratyphi), is a common cause of morbidity in the world, especially in South and Southeast Asia [1–3]. In 2015, an estimated 17 million cases of typhoid and paratyphoid fever occurred globally, mostly in South and Southeast Asia and sub-Saharan Africa. Typhoid and paratyphoid fever may be fatal if left untreated with an estimate of 178,000 deaths worldwide in 2015 [4, 5].

The global burden of Disease Study estimated that in 2017, 14.3 million cases of typhoid and paratyphoid fever occurred globally, where 12.6% of the cases occurred in children below 5 years and 55.9% occurred in children younger than 15 years of age [6]. A recent study conducted in Asia and Africa aimed to compare the proportion of children with enteric fever in the age groups < 5 years, 5–9 years and 10–14 years. The proportion of typhoid cases found in < 5 years age group ranged from 14 to 29%, in those 5–9 years of age the range was 30 to 44% and in 10–14 years of age the range found was 28 to 52% [7, 8]. School-aged children above 5 years of age have been reported to have high rates of enteric fever with incidence rates up to four times higher than adult populations [8, 9].

The highest incidence of typhoid fever was found in impoverished, overcrowded regions with poor sanitation such as urban slum areas of North Jakarta (Indonesia), Kolkata (India) and Karachi (Pakistan) [1, 10]. The persistence of typhoid in many low- and middle-income countries in Asia and Africa is due to contamination of water supply by human waste [11, 12].

The symptoms of typhoid fever are highly variable which may be mild, characterized by low grade fever and malaise. It may however cause severe life-threatening systemic infection with multiple complications such as intestinal perforation, intestinal haemorrhage and encephalopathy [13]. Typhoid treatment consists of antibiotics; however, an increasing number of drug-resistant strains have been found in endemic countries that prolong treatment and make it costly [14].

Improvement in sanitation and provision of clean water contributed to a decline in typhoid fever cases in developing countries during the early twentieth century. However, this change was not significant in countries where typhoid fever remains endemic and antibiotic resistance is on the rise. Vaccinations play an important part in the measure to reduce the burden of disease [15, 16].

Current available typhoid vaccines, parenteral Vi-polysaccharide (Vi-PS) and live oral Ty21a are not licensed for infants and toddlers. Vi-PS vaccines are poorly immunogenic in children below 2 years whereas Ty21a is currently available only in enteric-coated capsules, which makes it impractical for infants and toddlers [14, 17, 18]. These existing vaccines can provide 50–70% protection for 3–5 years in individuals over 2 years of age, however considering the above shortcomings, the necessity for the development of Typhoid Conjugate Vaccines (TCVs) was felt. The 2017 WHO Strategic Advisory Group of Experts on Immunization (SAGE) recommended the introduction of TCVs for infants and children aged > 6 months in typhoid endemic countries [3, 19]. TCVs provide longer- lasting protection, higher efficacy, require fewer doses and are suitable from infancy that allows them to be incorporated into the routine immunization program [15, 20].

An example of TCV is Typbar-TCV (Vi-polysaccharide conjugated to Tetanus toxoid), manufactured by Bharat Biotech International Limited, which through their 3- phase clinical trial, proved that it was safe, well tolerated and induced a robust and long-lasting response across age groups for long periods of time [17, 20, 21]. In addition to Typbar-TCV, which is a WHO prequalified vaccine, there are 3 other TCVs licensed in India. A Vi- tetanus toxoid conjugate vaccine from PedaTyph which showed satisfactory results with significant immunogenicity post vaccination [14]. The first prototype TCV was developed by US NIH and they conjugated Vi to recombinant exotoxin A of *Pseudomonas aeruginosa* (Vi-rEPA vaccine) that has been proven to produce a strong immune response in infants and toddlers [7, 17]. A clinical trial in Philippines studied the safety and immunogenicity of a vaccine with Vi- polysaccharide conjugated to Diphtheria toxoid (Vi-DT). Their phase I trial was conducted on subjects 2–45 years of age and their phase II trial was conducted on subjects 6 to 23 months. Both these phases of clinical trial proved that Vi-DT is safe, well-tolerated and immunogenic for the above age groups [22, 23].

This paper is a continuation of a previously published Phase I study involving subjects aged 2–5 years and 18–40 years as well as Phase II study involving subjects 6 to < 24 months [24, 25]. Although the phase II trial in subjects 2 to 11 years and 6 to < 24 months were held at the same time, the reports of these two age groups are being published separately due to some differences. First, there is no licensed Typhoid vaccine for children below 2 years in Indonesia, hence the control used in this age group was inactivated poliovirus vaccine whereas in children 2–11 years, the control used was an already licensed Vi-PS vaccine. Second, our phase I trial did not include children below 2 years therefore extra care had to be taken in this age group with 2 additional visit conducted, which was not the case in other age groups. Third, the objective of the trial in 6 to < 24 months group was safety and immunogenicity of Vi-DT vaccine whereas the objective of the trial on children 2–11 years was to compare safety and immunogenicity of Vi-DT to an already licensed vaccine. The results of the Phase I trial and phase II trial in children 6 to < 24 months proved that Vi-DT vaccine is safe with mild to moderate adverse effects and immunogenic with a significant increment in antibody GMT post vaccination. Hence, this study aims to evaluate the safety and immunogenicity of Vi-DT vaccine in children 2 to 11 years of age.

## Methods

### Study design

This study used a randomized, observer-blind, superiority design of Vi-DT vaccine compared to Vi-PS. A total of 200 children 2–11 years old were divided into 2 groups: half of them received Vi-DT and the other half Vi-PS.

### Sample size

The maximum seroconversion rate among controls was assumed as 0.7. If the true seroconversion rate for Vi-DT vaccine subjects is 0.9, the study required 82 subjects each in Vi-DT and Vi-PS groups to be able to reject the null hypothesis that the seroconversion rates for experimental and control subjects are equal, with probability of 0.9. The Type I error probability associated with two sided test of this null hypothesis is 0.05. By assuming a 20% dropout and issues related to inadequate samples, we enrolled 100 subjects in each group.

### Procedure

Inclusion criteria of this study were: healthy subjects age 2–11 years, parents or legal guardians agreed to abide by the rules of the study and visit schedule and signed the informed consent form.

Exclusion criteria were: subjects enrolled in another trial; had an axillary temperature of ≥37.5 °C; had a known history of allergy to any component of the vaccine; had a history of uncontrolled coagulopathy and receipt of treatment likely to alter immune response such as immunoglobulins, corticosteroids or other immunosuppressants. Subjects having an abnormality or chronic disease and subjects who previously suffered from typhoid fever (confirmed by blood culture or rapid test) were also excluded. Other exclusion criteria such as previous vaccination against typhoid fever; subjects already vaccinated with any vaccine within 1 month prior to vaccination or were expected to receive other vaccines within 1 month following vaccination and subjects who were planning to shift from the study area before the completion of the study.

After checking inclusion and exclusion criteria, the 200 subjects were recruited in such a way that 100 subjects received the experimental vaccine (Vi-DT) and 100 subjects received the control vaccine (Vi-PS). This allocation of groups was done by an unblinded team by giving the subject random codes which were unknown to the blinded investigators who were involved in recruitment, thereby, ensuring a non- biased result. The subjects were recruited by a team of blinded investigators from the Department of Child Health, Faculty of Medicine, Universitas Indonesia, Dr. Cipto Mangunkusumo General National Hospital, Jakarta.

The trial consisted of 2 visits to a primary health center: Visit 1 and Visit 2. During Visit 1, a pre-vaccination blood sample was taken before which subjects were given a single dose of either Vi-DT or Vi-PS vaccine and immediate adverse events were evaluated after vaccination. Subjects were given diary cards where they had to note down their daily temperature and list all the local and systemic reactions that occurred up to the second visit as well as the severity and duration of each reaction. During visit 2 (28 days post vaccination), subjects were followed up on adverse events and a second blood sample was taken to evaluate increment in antibody titers. These visits were carried out in Senen (Central Jakarta) primary health center and Jatinegara (East Jakarta) primary health center.

### Study intervention

The test vaccine used was Vi-DT vaccine produced by BioFarma which was injected intramuscularly in the left deltoid region. Each dose of this vaccine (0.5 mL) is composed of 25 μg of a purified Vi capsular polysaccharide of *S.* Typhi*,* 5 mg of 2-phenoxyethanol as preservative and 0.5 mL of phosphate buffer solution.

The control vaccine used was Vi-PS vaccine (Typhim Vi® produced by Sanofi), which is a licensed Typhoid vaccine in Indonesia. It was also injected intramuscularly in the left deltoid region. Each dose of this vaccine (0.5 mL) is composed of 25 µg of purified Vi polysaccharide in a colorless isotonic phosphate buffer saline (pH 7±0.3), 4.150 mg of Sodium Chloride, 0.065 mg of Disodium Phosphate, 0.023 mg of Monosodium Phosphate, Phenol 0.25%, and 0.5 ml of sterile water for injection.

### Safety evaluation

Immediate adverse effects (30 min post vaccination) were evaluated at visit 1. Delayed adverse effects (within 28 days post vaccination) were evaluated at visit 2. All local and systemic reactions, both immediate and delayed were recorded. This safety data was reviewed by a Data Safety Monitoring Board (DSMB). Vaccine safety data was analyzed using SPSS.

### Immunogenicity evaluation

The blood obtained at each visit was shipped to a Clinical Trial Laboratory of BioFarma where antibody titers were blindly tested using ELISA. Anti-Vi IgG titers were determined based on the international standard serum, NIBSC 16/138 recommended by the WHO Expert Committee on Biological Standardization [26].

The antibody increment and GMT 28 days post immunization were evaluated. A four-fold increase of antibody titer compared to the baseline value, was considered as the measure of seroconversion. Immunogenicity data was analyzed using SPSS.

Pre vaccination titer levels for subjects with zero titer level were assigned a value of 0.000001 to enable GMT and titer increment calculations. Post vaccination titer levels increased significantly, hence no values were assigned for these.

## Results

Two hundred subjects were recruited from October to December 2018 and followed up to 28 days post vaccination. There were no dropouts hence all 200 subjects were included in the data analysis. There were 104 females and 96 males. The mean age of subjects was 6.61 years for the Vi-DT group and 7.13 years for the Vi-PS group.

### Safety

Overall, adverse reactions between Vi-DT and Vi-PS were similar except for a significant difference in delayed systemic reactions 72 h to 28 days post vaccination where these reactions were found higher in the Vi-PS group (Table 1). Up to 24 h post vaccination, pain was the most common local reaction followed by redness. Fever and muscle pain were the most common systemic reactions. Both Vi-DT and Vi-PS groups had roughly the same number of adverse reactions and hence there was no significant *P* value (Table 2). Most cases were of mild to moderate intensities except for some severe cases of redness, swelling, induration and fever from 31 min to 72 h. Some severe cases of fever were also found from 72 h to 28 days post vaccination (Table 3). However, a majority of all cases, immediate and delayed, resolved within 48 h. There were no serious adverse events found up to 28 days post vaccination.

**Table 1:** Total number of local and systemic reactions 30 min to 28 days post vaccination

Description	Vi-DT (%) (*n* = 100)	Vi-PS (%) (*n* = 100)	***P*** value
**Immediate reactions (within 30 min)**			
Immediate local reactions	7	6	0.774
Immediate systemic reactions	2	1	1.00
**Delayed reactions (31 min - 24 h)**			
Delayed local reactions	10	13	0.056
Delayed systemic reactions	8	8	1
**Delayed adverse reactions (24–48 h)**			
Delayed local reactions	0	0	1
Delayed systemic reactions	1	2	1
**Delayed adverse reactions (48–72 h)**			
Delayed local reactions	0	0	1
Delayed systemic reactions	2	2	1
**Delayed adverse reactions (72 h-28 days)**			
Delayed local reactions	0	0	1
Delayed systemic reactions	5	18	0.003*

* Denotes a significant *p* value of < 0.05

**Table 2:** Local and systemic reactions 30 min to 24 h post vaccination

Description	30 min post vaccination			31 min to 24 h post vaccination		
	Vi-DT	Vi-PS	***P*** value	Vi-DT	Vi-PS	***P*** value
Pain	6%	6%	1	6%	7%	0.774
Redness	0	1%	1	4%	1%	0.386
Swelling	0	0	–	2%	2%	1
Induration	0	0	–	1%	0	1
Other local reactions	0	0	–	0	0	–
Fever	0	0	–	4%	4%	1
Fatigue	1%	1%	1	1%	0	1
Muscle pain	0	1%	1	3%	4%	0.976
Other systemic reactions	0	0	–	0	0	–

**Table 3:** Intensities of local and systemic reactions

	Within 30 min						*p*	31 min to 72 h						*p*	< 72 h to 28 days						*P*
	Vi-DT			Vi-PS				Vi-DT			Vi-PS				Vi-DT			Vi-PS			
	Mild	Moderate	Severe	Mild	Moderate	Severe		Mild	Moderate	Severe	Mild	Moderate	Severe		Mild	moderate	Severe	mild	Moderate	severe	
**Local Reaction**																					
Pain	6	0	0	6	0	0	1	6	0	0	7	0	0	1	0	0	0	0	0	0	–
Redness	0	0	0	1	0	0	1	2	0	2	1	0	0	1	0	0	0	0	0	0	–
Swelling	0	0	0	0	0	0	–	0	0	2	2	0	0	0.497	0	0	0	0	0	0	–
Induration	0	0	0	0	0	0	–	0	0	1	0	0	0	1	0	0	0	0	0	0	–
Other	0	0	0	0	0	0	–	0	0	0	0	0	0	–	0	0	0	0	0	0	–
**Systemic Event**																					
Fever	0	0	0	0	0	0	–	6	0	0	2	1	3	1	9	2	3	2	2	1	0.029
Fatigue	1	0	0	1	0	0	1	1	0	0	0	0	0	1	1	1	0	0	0	0	0.498
Muscle pain	0	0	0	1	0	0	1	2	1	0	4	0	0	1	0	0	0	0	0	0	–
Other	0	0	0	0	0	0	–	1	0	1	0	0	0	0.498	0	0	0	0	0	0	–

### Immunogenicity

Anti-Vi IgG antibody titers were measured pre-vaccination and 28 days post vaccination. GMT at 28 days post vaccination drastically increased, with a significantly higher value in Vi-DT compared to Vi-PS (*p* < 0.001) (Table 4). At 28 days post vaccination, 100% of the subjects in the Vi-DT group experienced antibody increment ≥4-fold, whereas 93% of the subjects in the Vi-PS group experienced antibody increment ≥4-fold (Table 5).

**Table 4:** Geometric mean titer (GMT) of antibody following vaccination

Vaccine	GMT V1 (95%CI) Pre- vaccination	***p***	GMT V2 (95%CI) 28 days post vaccination	***P***
**Vi-DT**	0.0001445	0.224	185.1685	< 0.001
**Vi-PS**	0.0001281		55.534402

**Table 5:** Percentage of subjects with increasing antibody titer 28 days post vaccination

Group	Increment of antibody titers V1 to V2		***p***	RR (95%CI)
	< 4-fold	≥ 4-fold		
**Vi-DT**	0	100%	0.014	0.930 (0.881–0.981)
**Vi-PS**	7%	93%		

## Discussion

This phase II trial on subjects 2–11 years is a continuation of a previously published phase I trial [24]. In this age group, the Vi-DT conjugate vaccine resulted in minimal local and systemic adverse reactions. This vaccine also induced antibody responses in 100% of subjects with GMT titers significantly higher than Vi-PS vaccine. Thus, safety and immunogenicity of Vi-DT is now established for children 2–11 years.

The phase I trial was conducted on subjects 2–5 years and 18–40 years old [24]. This trial concluded that Vi-DT vaccine is safe, with minimal adverse effects and a single dose could produce a significant increment in anti-Vi IgG antibody levels. Based on these results, we decided to evaluate the safety and immunogenicity in children 2–11 years old. There were no dropouts, hence all 200 subjects participated in visit 1 and visit 2 and all were available for data analysis.

Previously, phase I randomized controlled trial of another Vi-DT vaccine (SK bioscience, South Korea) was conducted in the Philippines to evaluate safety and immunogenicity of a Vi-DT vaccine compared to Vi-PS vaccine in subjects 2–45 years old [22]. Pain at injection site was the most common immediate local reaction whereas fever was the most common immediate systemic reaction found in children. All cases were of mild to moderate severities except for one case of severe fever. There were no serious adverse events [22]. Our study is similar to this study in Philippines in regard to adverse reactions. We found that pain was the most common local reaction. Fever and muscle pain were the most common systemic reactions. There was no significant difference in reaction between Vi-DT and Vi-PS. Most cases were of mild to moderate intensities, except for some cases of severe fever found 72 h to 28 days post vaccination. These events were caused by bacterial or viral infections and resolved within 48 h without any complications. Similar to the Philippines study, our study did not have any serious adverse events up to 28 days post vaccination [24].

Previous phase 1 study of the Vi-DT vaccine (SK bioscience) conducted in Philippines showed that 100% of subjects in the Vi-DT group whereas 97% of subjects in the Vi-PS group showed seroconversion respectively. The Vi-DT group had a significantly higher GMT than the Vi-PS group (*p* < 0.001) [22]. Our trial also showed similar results where 100% of the subjects in the Vi-DT group showed antibody increment of ≥4 fold whereas 93% of subjects in the Vi-PS group showed increment of ≥4 fold as compared to baseline. GMT at 28 days post vaccination was also significantly higher in the Vi-DT group as compared to the Vi-PS group (*p* < 0.001) [24].

A randomized study in India aimed to evaluate safety and immunogenicity of a Vi- tetanus toxoid vaccine (PedaTyph™) in subjects 6 months to 12 years. They were given two doses of vaccines 6 weeks apart. Like our trial, this trial also had pain and fever as the most common adverse reactions. Identical to our trial, 100% of the subjects in this trial exhibited seroconversion at 6 weeks post vaccination [27].

Another randomized controlled study to evaluate the safety and immunogenicity of a Vi polysaccharide- tetanus toxoid vaccine (Typbar TCV) was carried out in India. This trial involved subjects 2 to 45 years. Similar to our trial, the most common adverse reaction found was fever. 97.3% of subjects who received TCV and 93.1% of subjects who received control (Vi-polysaccharide Typbar) exhibited seroconversion. This value of seroconversion was found higher in our trial where 100% of the subjects who received Vi-DT TCV exhibited seroconversion. Like our trial, this trial had significant GMT increment (*p* < 0.001) [17].

When compared to the phase II trial on children 6 to < 24 months, both these age groups had pain as the most common local reaction and fever as well muscle pain as the most common systemic reactions. Antibody increment for Vi-DT was slightly higher in 2–11 years age group compared to 6 to < 24 months group (100 and 98.99% respectively). GMT for both groups increased significantly [25].

Based on the safety and immunogenicity described in this study and previously published papers, it can be concluded that the typhoid conjugate Vi-DT vaccine is safe and immunogenic.

## Conclusion

Our phase II study concluded that the novel typhoid conjugate Vi-DT vaccine is safe and immunogenic in children 2–11 years old.

## Data Availability

The datasets generated and analyzed during the current study are not publicly available because this a study on a new vaccine which is still ongoing and authors have a responsibility to the funding source to limit the data shared. However, these are available from the corresponding author on reasonable request. This manuscript adheres to CONSORT guidelines.

## Abbreviations

*S. Typhi*: Salmonella Typhi
Vi-DT: Vi polysaccharide conjugated to diphtheria toxoid
Vi-PS: Vi Polysaccharide
TCV: Typhoid conjugate vaccine
IgG: Immunoglobulin G
GMT: Geometric mean titer
WHO: World Health Organization
SAGE: Strategic Advisory Group of Experts on Immunization
Vi-rEPA: Vi conjugated to *Pseudomonas aeruginosa*
ELISA: Enzyme-linked immunosorbent *assay*
